# Stable distance regression via spatial–frequency state space model for robot-assisted endomicroscopy

**DOI:** 10.1007/s11548-025-03353-w

**Published:** 2025-04-12

**Authors:** Mengyi Zhou, Chi Xu, Stamatia Giannarou

**Affiliations:** Hamlyn Centre for Robotic Surgery, Department of Surgery and Cancer, Imperial College London, London, SW7 2AZ UK

**Keywords:** Distance regression, State space model, Fine-tuning, Robotic tissue scanning, Endomicroscopy

## Abstract

**Purpose:**

Probe-based confocal laser endomicroscopy (pCLE) is a noninvasive technique that enables the direct visualization of tissue at a microscopic level in real time. One of the main challenges in using pCLE is maintaining the probe within a working range of micrometer scale. As a result, the need arises for automatically regressing the probe–tissue distance to enable precise robotic tissue scanning.

**Methods:**

In this paper, we propose the spatial frequency bidirectional structured state space model (SF-BiS4D) for pCLE probe–tissue distance regression. This model advances traditional state space models by processing image sequences bidirectionally and analyzing data in both the frequency and spatial domains. Additionally, we introduce a guided trajectory planning strategy that generates pseudo-distance labels, facilitating the training of sequential models to generate smooth and stable robotic scanning trajectories. To improve inference speed, we also implement a hierarchical guided fine-tuning (GF) approach that efficiently reduces the size of the BiS4D model while maintaining performance.

**Results:**

The performance of our proposed model has been evaluated both qualitatively and quantitatively using the pCLE regression dataset (PRD). In comparison with existing state-of-the-art (SOTA) methods, our approach demonstrated superior performance in terms of accuracy and stability.

**Conclusion:**

Our proposed deep learning-based framework effectively improves distance regression for microscopic visual servoing and demonstrates its potential for integration into surgical procedures requiring precise real-time intraoperative imaging.

**Supplementary Information:**

The online version contains supplementary material available at 10.1007/s11548-025-03353-w.

## Introduction

In recent years, pCLE has emerged as a SOTA biophotonics technique that enables real-time visualization of tissue cellular morphology. This noninvasive imaging method has demonstrated great potential for tissue characterization during tumor resection, greatly enhancing the precision and effectiveness of oncological surgeries, such as open neurosurgery [1–3]. However, maintaining the ideal working distance between the tissue and the pCLE probe, which is of micrometer scale, presents a significant ergonomic challenge during manual tissue scanning. Precise scanning can be achieved through microsurgical robotic manipulation and automatic estimation of the probe–tissue distance and orientation has attracted significant interest recently [4–8].

The field of medical imaging has experienced significant advancements with the integration of deep learning technologies, particularly in developing regression networks aimed at automating the focusing process in confocal microscopy. Research in this area has led to a variety of innovative approaches. For example, Zhenbo et al. [9] and Tomi et al. [10] adapted conventional convolutional neural network (CNN) models to accurately predict the optimal focus distance for capturing in-focus images. Diverging from methods reliant on spatial domain information, Jiang et al. [11] introduced a novel preprocessing technique that converts images into a multi-domain representation, subsequently fed to a regression CNN model to enhance focusing accuracy. Similarly, Zhang et al. [12] employed Sobel filters in various orientations to produce gradient images. These images serve as inputs to a diversity-learning network, which determines the precise focus distance.

Maintaining the pCLE probe within its working range has proven to be more challenging than traditional confocal microscopy, because improvements in image clarity become less apparent as we approach the optimal position [8]. This is in contrast to the more linear relationship observed in conventional microscopy methods. Furthermore, the pronounced noise in pCLE data complicates accurate distance estimation with deep learning networks. To address these challenges, Xu et al. developed the SFFC-Net, which leverages both frequency and spatial domain features to enhance distance regression accuracy [5]. Additionally, they developed a generative adversarial network (GAN) and sequence-attention (SA) module [6] to incorporate robust image-based supervision and temporal information, respectively. However, attention-based modules can easily memorize the training data rather than learn generalizable patterns for short image sequences [13]. This may affect the stability of the regression.

In the literature, temporal models, like recurrent neural networks (RNNs), have been proposed for the analysis of sequential images, being able to utilize temporal information effectively [14–16]. The recently proposed state space model (SSM) provides a parametric framework for mapping input information to output predictions, being suitable for processing time-series data across various domains [17]. Notably, Gu et al. developed the structured state space (S4) model [18] and its variant Mamba [19], which have shown superior efficiency in extracting temporal information from sequential data.

In this work, we designed and implemented a regression framework for visual servoing in robot-assisted pCLE tissue scanning. (1) To enforce stability during scanning, we propose a novel distance regression method which fuses spatial–frequency and temporal information. To learn temporal information, different from the sequence-attention layer proposed in [6], we developed the bidirectional structured state space model (BiS4D) which advances the SSM by incorporating bidirectional image sequence processing and analyzing data representations in both the frequency and spatial domains to improve the stability of model regression. (2) To enable the sequential model to learn smooth and stable probe trajectories for robotic scanning, instead of using the ground truth distance labels [5, 6, 14–16], a novel guided trajectory planning strategy is designed to generate pseudo-distance labels for sequential model training. (3) To speed up the inference time, the hierarchical guided fine-tuning approach is introduced to effectively reduce the size of the BiS4D model and maintain the performance. The proposed method has been extensively validated on *ex vivo* pCLE data and has shown superior performance in terms of stability and accuracy.

## Methodology

**Fig. 1:**
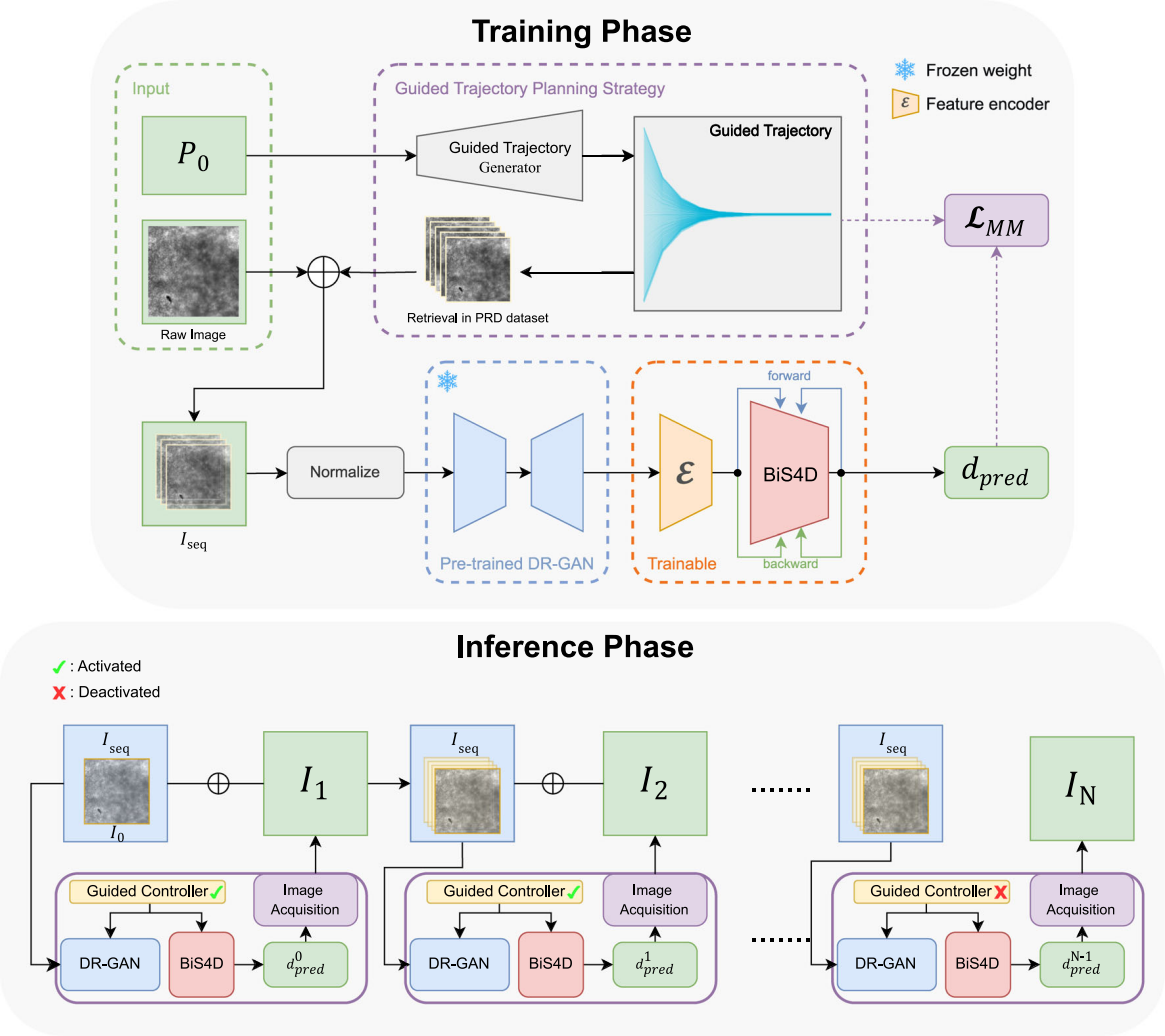
The overall framework of SF-BiS4D. (Top) The Training Phase. $\bigoplus$ represents the concatenation operation. Here, the raw image is concatenated with the image sequence $I_{seq}$ retrieved from the PRD dataset. $\mathcal {E}$ represents the feature encoder between the feature extractor and the BiS4D. (Bottom) The Inference Phase. If fewer than 10 frames are available, we use all the available frames

The proposed framework is composed of two distinct phases, namely, a training phase, where the model learns to predict probe–tissue distances, and an inference phase, where the model guides the probe to converge to the optimal scanning position and capture pCLE data.

As shown in Fig. 1, during the training phase, each input contains the initial probe position $P_0$ relative to the tissue surface and the corresponding pCLE image. Initially, the guided trajectory generator $\mathcal {G}_T(\cdot )$ uses $P_0$ to produce a series of positions, leading to the optimal scanning position, forming the guided trajectory $\textbf{P}_{seq} = \mathcal {G}_T(P_0)$. Corresponding pCLE images $\textbf{I}_{seq}$ from these positions are then extracted from the PRD dataset video, normalized, and fed into the SF-BiS4D model. The pretrained DR-GAN $\mathcal {F}_{FE}(\cdot )$ [6] extracts spatial and frequency domain features from each image ($u_i = \mathcal {F}_{FE}(\textbf{I}^i_{seq})$). These features are concatenated into feature sequences $\textbf{U}_{seq}$ and processed by the encoder $\varvec{\varepsilon }(\cdot )$ for feature fusion. The BiS4D model $\mathcal {F}_{T}(\cdot )$ then analyzes the fused features to predict the probe–tissue distance for each sequence image ($d_{pred} = \mathcal {F}_{T}(\varvec{\varepsilon }(\textbf{U}_{seq}))$), using a loss function $\mathcal {L}_{MM}(d_{pred}, d_{GT})$ to minimize the error between predicted and ground truth distances.

In the inference phase, the trained SF-BiS4D model analyzes the initially acquired pCLE image $I_0$ to predict the probe–tissue distance $d^0_{pred} = \mathcal {F}_{T}(\varvec{\varepsilon }(\mathcal {F}_{FE}(I_0)))$. Based on $d^0_{pred}$, the position of the probe is adjusted with respect to the tissue surface, and a new pCLE image $I_1$ is acquired and added to the sequence $\textbf{I}_{seq}$ for subsequent distance prediction. This process is repeated until convergence (when the probe is predicted to be stabilized within the working range), as outlined in the inference phase in Fig. 1.

### Spatial frequency bidirectional structured state space model (SF-BiS4D)

To extract efficient pCLE data representations, the DR-GAN feature extractor is used to generate spatial and frequency domain features (SF). Frequency features are important as they contain information related to image blurriness and therefore to the distance of the probe from the tissue surface (i.e., blurry images are associated with high probe–tissue distance and contain more low-frequency information) [5].

To regress the distance between the pCLE probe and the tissue surface, we designed the BiS4D model. Our model extends the S4D model [18] to process an image sequence in both forward and backward directions. This enables the model to capture data dependencies not only from past to future but also from future to past. To achieve this, two parallel stacks have been created, namely, the forward S4D layer and the backward S4D layers. Image sequences are processed in both forward and reverse order separately by these two stacks.

**Fig. 2:**
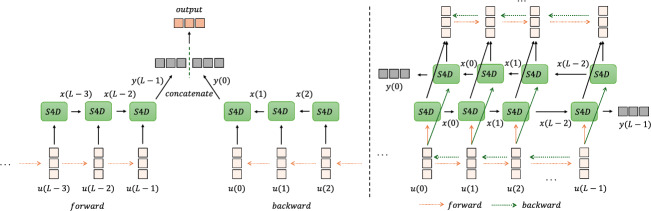
The structure of BiS4D. (Left) A single bidirectional layer BiS4D. (Right) The schematic diagram of the multilayer BiS4D network

The structure of a single bidirectional layer BiS4D is depicted in Fig. 2 (Left). For a sequence of image features $\textbf{U}_{seq}$ of length *L*, the forward S4D branch sequentially processes the input. Conversely, the backward S4D branch processes the reverse feature sequence $\overleftarrow{\textbf{U}_{seq}}$, thereby capturing temporal dependencies in the opposite direction. Subsequently, the model combines the results of both branches and resizes it to make it of the same size as the input of the regressor.

To improve the accuracy of the regression, multiple forward and backward S4D layers can be integrated as depicted in Fig. 2 (Right) and described in Algorithm 1. The output of forward and backward S4D modules at each layer is integrated sequentially, and subsequently fed to the next layer. In Algorithm 1, the $\textbf{U}_{fwd}$ and $\textbf{U}_{bwd}$ are forward and backward sequential features, respectively.

**Algorithm 1:**
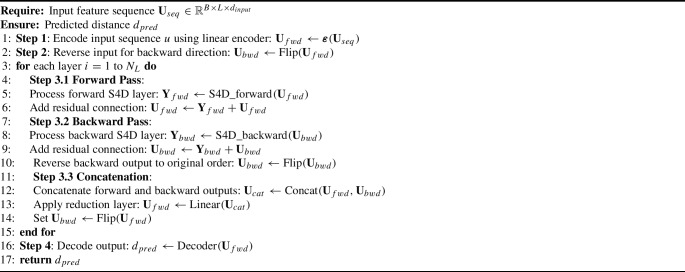
Bidirectional S4D Algorithm

### Guided trajectory planning strategy

The aim of this component is to generate pairs of pCLE images and pseudo-distance labels which simulate smooth probe trajectories. This will enable our sequential model BiS4D to learn smooth and stable probe trajectories for robotic scanning which will enable the probe to converge to the optimal position within the working range. For this purpose, we propose a guided distance trajectory planning strategy, the so-called exponential distance discretization (EDD).

**Exponential Distance Discretization** For a given initial position $P_0\in [-400\mu m,400\mu m]$, we generate a sequence of positions $P_i$ $(i =0,1,...,N)$ which represent probe distances with respect to the tissue surface. These distances are distributed exponentially across *N* steps and calculated as:

1 $$\begin{aligned} P_i = P_{\text {0}} \cdot \exp {\left( \frac{i}{N-1} \cdot \ln \left( \frac{P_{\text {0}}}{\alpha }\right) \right) } \end{aligned}$$

where $\alpha$ is a hyperparameter which controls the speed of the convergence to the optimal position, which is set to 0.1 for fast convergence.

However, Eq.(1) only includes calculations for which $P_0 > 0$. Consequently, to accommodate both for positive and negative positions, $P_i$ is generated as:

2 $$\begin{aligned} P_i = {\left\{ \begin{array}{ll} -|P_0| \cdot \exp {\left( \frac{i}{N-1} \cdot \ln \left( \frac{|P_0|}{\alpha }\right) \right) } &  \text {if } P_0 < 0, \\ 0 &  \text {if } P_0 = 0, \\ P_{0} \cdot \exp {\left( \frac{i}{N-1} \cdot \ln \left( \frac{P_{0}}{\alpha }\right) \right) } &  \text {if } P_0 > 0. \end{array}\right. } \end{aligned}$$

Subsequently, the generated position sequences are discretized by rounding them to the nearest multiple of 5 as in Eq.(3), ensuring compatibility with the ground truth distance labels which have been generated using a distance step equal to $5\mu m$.

3 $$\begin{aligned} P_i^{discretized}=\left\lfloor \frac{P_i}{5}\right\rfloor \cdot 5 \end{aligned}$$

where the operator $\lfloor \cdot \rfloor$ represents the rounding down function.

### Hierarchical guided fine-tuning

To mitigate the computational complexity of the BiS4D model, we designed a fine-tuning approach. This involves reducing the number of bidirectional layers to 1. To preserve performance despite this layer reduction, we also incorporated the pretrained DR-GAN [6] as a frozen feature extractor, while fine-tuning the encoder $\varvec{\varepsilon }$ within the BiS4D model. Although this modification, namely, F-SF-BiS4D, maintained performance of stability, it significantly slowed the convergence rate, as evidenced by the MAE$_{1st}$ in Table 3.

In response to this challenge, we designed the guided controller, which controls the activation of the DR-GAN regressor and the deactivation of the F-SF-BiS4D model during the inference phase. Initially, in the absence of substantial temporal data, such as at the early stages of tissue scanning, the guided controller (G) utilizes the DR-GAN regressor to predict the probe–tissue distance for the first three scanning steps. The use of the DR-GAN regressor allows the model to converge to the valid working range rapidly. Once sufficient temporal information becomes available after the initial 3 steps, the guided controller is deactivated, allowing the F-SF-BiS4D to operate. This hierarchical inference design ensures that the guided fine-tuned SF-BiS4D model (GF-SF-BiS4D) integrates both guided initialization and fine-tuning, optimizing both speed of convergence and stability.

### Loss function

To train our proposed regressor, the model has been optimized utilizing the mean absolute error (MAE) and mean absolute percentage error (MAPE), in our loss function as $\mathcal {L}_{MP}=$ $\frac{1}{2}\cdot$(MAE+MAPE).

**MAE** The MAE is a simple and robust loss function calculated as:

4 $$\begin{aligned} \textrm{MAE} = \frac{1}{n}\sum _{i = 1}^{n}|y_i - \hat{y_i}|\end{aligned}$$

where *n* is the number of data, $y_i$ is the true value, and $\hat{y_i}$ is the predicted value.

**MAPE** In situations where the distance between the pCLE probe and the tissue surface is within the optimal imaging range, it could be desirable for the model to remain within this range and to predict the distance with the greatest possible accuracy. Thus, we use the MAPE defined as:

5 $$\begin{aligned} \textrm{MAPE} = \frac{1}{n}\sum ^n_{i=1}|\frac{y_i-\hat{y_i}}{y_i} |\times 100 \end{aligned}$$

## Experiments

**PRD Dataset**^1^ [5]. *ex vivo* pig brain tissue, treated with 0.1% acriflavin, was scanned using a Z 1800 confocal miniprobe (Cellvizio, Mauna Kea Technologies, Paris). The miniprobe was operated using the Kinesis^®^ K-Cube™ Stepper Motor Controller (Thorlabs, USA), covering a distance range of 400 $\mu$m to -400 $\mu$m from the tissue surface in steps of 5 $\mu$m. A total of 62 pCLE videos and their corresponding probe positions were recorded from independent samples. The optimal scanning position was identified by locating the pCLE frame with the least blur, confirmed through expert review by a neurosurgeon. According to the Z miniprobe’s specifications, its working range is between 35 $\mu$m and 400 $\mu$m from the tissue surface. For the experiment, 50 videos (7,539 frames) were used for training, while 12 videos (1,706 frames) were reserved for testing.

**Implementation details** All experiments were conducted on an NVIDIA RTX A5000 GPU with the memory of 24GB, based on the PyTorch framework. The batch size during training was set to 8, and the same random seed was employed across all experiments. The Adam optimizer [20] was employed with a cyclic learning rate schedule [21], where the learning rate oscillates between $1 \times 10^{-5}$ and $1 \times 10^{-4}$ every 5 epochs, helping the model achieve better gradient descent performance. All the images used in the training and inference phase have been normalized following the same local and global normalization method previously proposed in [5]. In the experiments, all models are trained on the training datasets. All trained models are frozen and validated on the testing datasets.

**Fig. 3:**
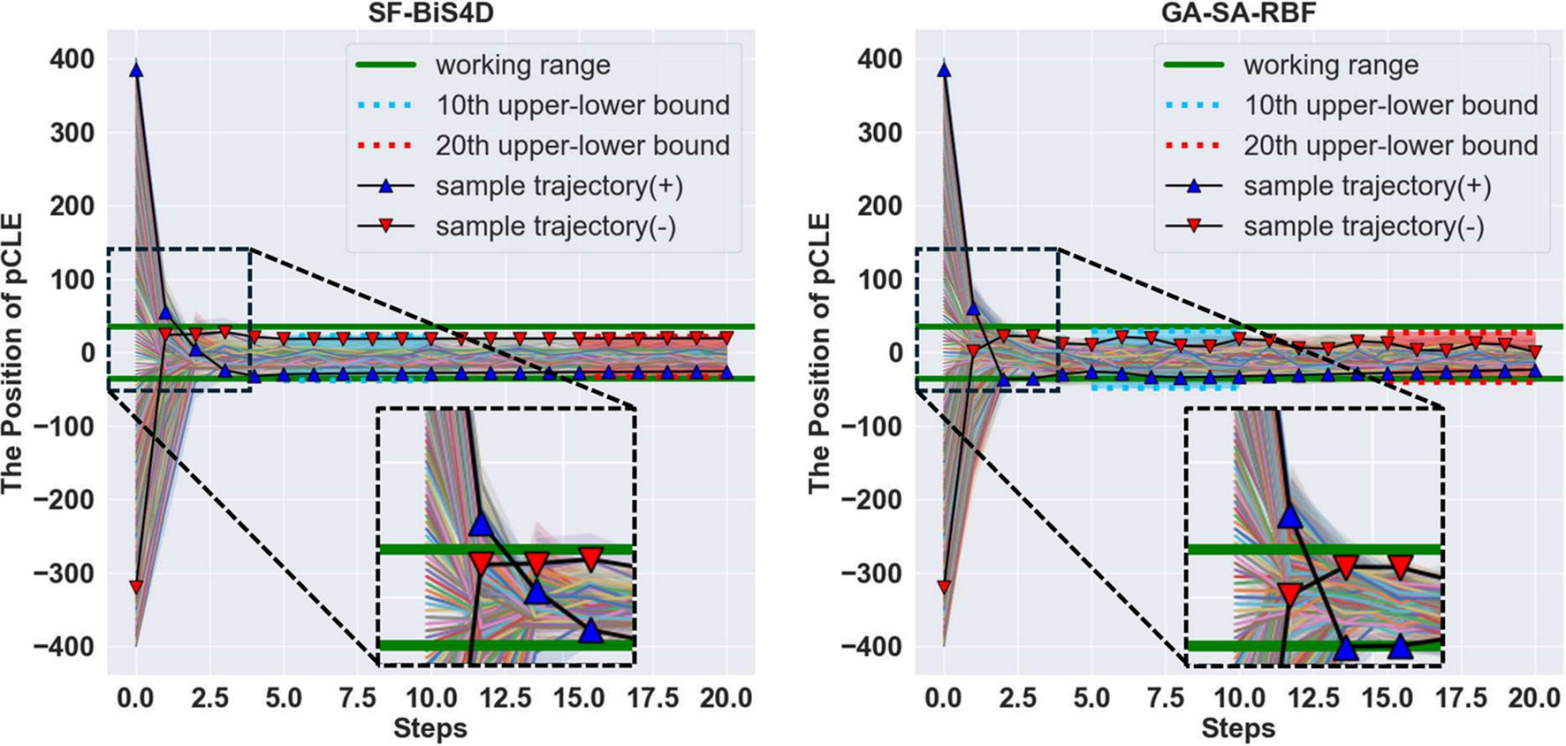
K-step incremental analysis of SF-BiS4D and GA-SA-RBF on the testing set. The figure illustrates the positions of the pCLE probe predicted by the regression models. Two different trajectories are presented for initial images taken from the test set, located at 385 $\mu$m and -320 $\mu$m which are presented as lines with red and blue triangles, respectively. Within the first four steps, the probe converges within the working range. As the probe can acquire high-quality images at this position, the model for the next steps stabilizes the probe at this position and predicts small subsequent movements

**Convergence and stability study** The convergence and stability study is aimed at validating whether the regression model can guide the robotic system to converge to the optimal scanning position and stabilize there during the robotic scanning. We followed the evaluation methods proposed in [5], using a K-step incremental analysis, with image feedback steps ranging from $k = 1$ to *K*. Each test pCLE image has a corresponding ground truth probe–tissue distance, which serves as the initial position $P_0$ for the K-step experiment. At each step *k*, the pCLE image is concatenated with the previous image sequence and fed to the model to predict the probe–tissue distance $d^k_{pred}$. The new probe position is then calculated based on the predicted distance. This process is repeated for 20 iterations and the resulting trajectories of the probe are analyzed using the evaluation metrics of convergence and stability. The quality of convergence is assessed by the metrics MAE$^C$ and *BM*, which are the mean absolute error and the blurriness scores of pCLE images estimated by blur metrics proposed by [6] after convergence, respectively. The stability of regression is evaluated by the width of the upper-lower bound after convergence (blue and red dotted lines in Fig. 3).

**Comparison study** To ensure consistency with established benchmarks proposed in [6], we evaluated the performance of our model using the same evaluation criteria defined in [6] , namely, MAE$_{1st}$, MAE$^C$, ACC$_{dir}$, $W^B$, and *BM*. Our model was compared against SOTA single-image distance regression models, SFFC-Net [5] and DR-GAN [6], as well as the temporal information-enhanced GA-SA-RBF model [6]. As depicted in Table 1, the SF-BiS4D with three bidirectional layers outperforms both SFFC-Net and DR-GAN in terms of MAE$_{1st}$ and ACC$_{dir}$, demonstrating faster convergence (also verified in Fig. 3). Moreover, when compared to the GA-SA-RBF model, both our SF-BiS4D and the GF-SF-BiS4D models show significantly improved performance in MAE$^C_{20}$, $BM_{20}$, and $W^B_{20}$. As shown in Table 2, a paired t test comparing SFFC-Net, DR-GAN, and GA-SA-RBF with our models (SF-BiS4D, GF-SF-BiS4D) showed p values significantly below 0.05 for all metrics with SFFC-Net and DR-GAN, confirming statistical significance. For GA-SA-RBF, p values were below 0.05 for MAE$^C_{20}$ and $W^B_{20}$, indicating our models’ superiority, while $BM_{20}$ showed comparable performance with near-zero *t*-statistic value. The stability of the trajectories produced by SF-BiS4D, as shown in Fig. 3, is notably higher than those from the GA-SA-RBF model. This indicates that our BiS4D sequential model is more effective at learning temporal information than the attention-based GA-SA-RBF model.

**Table 1:** Comparison of regression models. The unit of $$W^B $$, MAE$$_{1st}$$, and MAE$$^C$$ is $$\mu m$$. The *Params* are the overall parameters of the model and *t* is the inference time per frame (ms). The best results are highlighted in **bold**, and the second-best results are underlined

Network	MAE$_{1st} \downarrow$	ACC$_{dir} \uparrow$	MAE$^C_{20} \downarrow$	$BM_{20} \uparrow$	$W^B_{20} \downarrow$	$Params \downarrow$	*t*
SFFC-Net	64.29	94.49%	41.19 ± 0.91	0.905	113.94	**14 M**	**13**.**6**
DR-GAN	63.68	94.55%	32.99 ± 0.97	0.920	70.68	**14 M**	**13**.**6**
GA-SA-RBF	64.30	94.90%	31.11 ± 0.31	**0**.**941**	67.65	18.3M	18.3
SF-BiS4D	**63**.**57**	**95.43%**	$\underline{28.73\pm 0.17}$	**0**.**941**	55.59	17.1M	19.7
GF-SF-BiS4D	63.68	94.55%	${\textbf {25.32}}\pm {\textbf {0.07}}$	0.940	**50**.**96**	16.4M	15.7

**Table 2:** Paired t test of SOTA methods vs SF-BiS4D and GF-SF-BiS4D. "*t*-statistic" is the t-statistic value, and $p<0.05$ shows whether the condition "p value < 0.05" is satisfied to prove the statistical significance. $\checkmark$ means statistical significance and $\times$ means no statistical significance

Metrics	SFFC-Net		DR-GAN		GA-SA-RBF	
	*t*-statistic	$p<0.05$	*t*-statistic	$p<0.05$	*t*-statistic	$p<0.05$
***SOTA models vs SF-BiS4D***						
$\textrm{MAE}^{20}_{C}$	555.93	$\checkmark$	178.67	$\checkmark$	278.04	$\checkmark$
$BM_{20}$	21.06	$\checkmark$	38.79	$\checkmark$	0	$\times$
$W^{B}_{20}$	2603.39	$\checkmark$	632.90	$\checkmark$	1408.91	$\checkmark$
***SOTA models vs GF-SF-BiS4D***						
$\textrm{MAE}^{20}_{C}$	718.20	$\checkmark$	325.75	$\checkmark$	752.50	$\checkmark$
$BM_{20}$	21.87	$\checkmark$	40.64	$\checkmark$	1.85	$\times$
$W^{B}_{20}$	2850.16	$\checkmark$	837.52	$\checkmark$	2169.13	$\checkmark$

**Ablation Study** Ablation study was conducted to evaluate the contribution of different strategies to our regression model’s performance, using the DR-GAN as the baseline.

**Table 3:** Ablation study of the proposed strategies. GTP, FT, G represent guided trajectory planning, fine-tuning, and guided controller. The symbol $^\mathrm{w/o}$ represents the model trained without GTP. $\checkmark$ represents the inclusion of a strategy. The best results are highlighted in **bold**, and the second-best results are underlined

Network	BiS4D	GTP	FT	G	MAE$$_{1st}$$	ACC$$_{dir} $$	MAE$$^C_{20} $$	$$BM_{20}$$	$$W^B_{20}$$
DR-GAN					63.68	94.55%	32.99 ± 0.97	0.920	70.68
S-BiS4D	$\checkmark$				78.67	95.25%	30.40 ± 0.66	0.889	73.26
SF-BiS4D$^\mathrm{w/o}$	$\checkmark$				66.55	95.13%	30.26 ± 0.48	0.901	70.68
SF-BiS4D	$\checkmark$	$\checkmark$			**63**.**57**	**95.43%**	28.73 ± 0.17	0.941	55.59
F-SF-BiS4D	$\checkmark$	$\checkmark$	$\checkmark$		72.76	95.25%	25.46 ± **0**.**07**	0.932	**45**.**96**
G-SF-BiS4D	$\checkmark$	$\checkmark$		$\checkmark$	63.68	94.55%	28.82 ± 0.23	**0**.**942**	56.37
GF-SF-BiS4D	$\checkmark$	$\checkmark$	$\checkmark$	$\checkmark$	63.68	94.55%	${\textbf {25.32}}\pm {\textbf {0.07}}$	0.940	50.96

**Table 4:** Ablation study of hyperparameters and loss functions. **L** is the sequence length for BiS4D sequential model. ${{\textbf {N}}}_{S}$ is the number of steps when guided controller activated in GF-SF-BiS4D. $\mathcal {L}$ is loss function in training SF-BiS4D, where $\mathcal {L}_{M}$ is MAE, $\mathcal {L}_{P}$ is MAPE, $\mathcal {L}_{MP}$ is MAE+MAPE, and $\mathcal {L}_{L}$ is the likelihood loss function. The best results are highlighted in **bold**

	MAE$_{1st}$	ACC$_{dir}$	MAE$^C_{10}$	MAE$^C_{20}$	$BM_{10}$	$BM_{20}$	$W^B_{10}$	$W^B_{20}$
**L**								
5	72.51	**96.13%**	37.93 ± **0**.**28**	37.07 ± 0.17	0.895	0.896	88.38	85.24
10	**63**.**57**	95.43%	**29**.**66** ± 0.48	${\textbf {28.73}}\pm {\textbf {0.17}}$	**0**.**939**	**0**.**941**	**60**.**44**	**55**.**59**
15	66.47	94.96%	31.04 ± 0.52	30.52 ± 0.31	0.916	0.926	74.57	69.92
20	117.04	94.49%	36.42 ± 6.41	34.45 ± 0.22	0.906	0.917	144.90	90.28
$${\textbf {N}}_{S}$$								
0	72.76	**95.25%**	27.23 ± 0.94	25.46 ± 0.07	0.925	0.932	63.43	**45**.**96**
1	**63**.**68**	94.55%	27.82 ± 0.79	26.50 ± 0.11	0.930	0.935	57.86	56.42
2	**63**.**68**	94.55%	27.13 ± **0**.**50**	25.93 ± **0**.**06**	0.935	0.939	57.32	55.51
3	**63**.**68**	94.55%	**26**.**99** ± 0.82	**25**.**32** ± 0.07	**0**.**937**	**0**.**940**	**54**.**78**	50.96
4	**63**.**68**	94.55%	27.29 ± 1.25	25.77 ± 0.08	0.927	0.934	64.17	61.33
$$\mathcal {L}$$								
$\mathcal {L}_{M}$	81.10	**96.24%**	33.86 ± 1.30	30.42 ± 0.47	0.909	0.936	90.80	66.80
$\mathcal {L}_{P}$	67.10	94.95%	30.00 ± 0.61	28.16 ± 0.18	0.930	0.940	72.02	56.39
$\mathcal {L}_{MM}$	**63**.**57**	95.43%	${\textbf {29.66}}\pm {\textbf {0.48}}$	${\textbf {28.73}}\pm {\textbf {0.17}}$	**0**.**939**	**0**.**941**	**60**.**44**	**55**.**59**
$\mathcal {L}_{L}$	63.83	94.96%	29.88 ± 0.99	31.93 ± 1.26	0.917	0.924	73.26	72.86

As demonstrated in Table 3, integrating our BiS4D sequential model and guided trajectory planning (GTP) strategy into the pretrained DR-GAN feature extractor significantly enhances both convergence and stability (rows 4-6). To evaluate the impact of feature domains on distance regression, we compared models which use only spatial domain features (S-BiS4D) against those which incorporate both spatial and frequency domain features (SF-BiS4D) in rows 2 and 4. The results show that SF-BiS4D surpasses S-BiS4D across all performance metrics. Additionally, to understand the specific contribution of GTP, we compared the performance of the SF-BiS4D model with and without the GTP strategy (rows 3 and 4). The results indicate a significantly higher $BM_{20}$ and lower $W^B_{20}$ for SF-BiS4D with GTP, suggesting that this strategy not only stabilizes trajectories but also enhances the quality of pCLE images after convergence. Furthermore, rows 5–7 illustrate that the guided controller (G) accelerates convergence, while fine-tuning (FT) boosts stability.

We also conducted ablation experiments on the hyperparameters which include the sequence length **L** for sequential models and the number of steps **N**$_{S}$ after which the guided controller G is deactivated in GF-SF-BiS4D. Additionally, we compared various loss functions: the MAE loss ($\mathcal {L}_M$), MAE+MAPE loss ($\mathcal {L}_{MM}$), and likelihood-based mean squared error ($\mathcal {L_{L}}$) [22], as detailed in the final section of Table 4. Results in Table 4 indicate that the BiS4D sequential model exhibits optimal regression accuracy and stability with an input sequence length **L** of 10. For guided controller G, the GF-SF-BiS4D model has the highest stability of regression when the G is activated in the first three steps. Furthermore, the SF-BiS4D model trained by $\mathcal {L}_{MM}$ demonstrates superior performance in both accuracy and stability, as shown in the last section of Table 4.

## Conclusion

This paper introduces the spatial frequency bidirectional structured state space model (SF-BiS4D), a novel deep learning framework for guiding robot-assisted endomicroscopy with pCLE. Our performance evaluation study has shown that the proposed regression model can be deployed in real time and with accuracy which is within the valid working range. So far, the model has been trained on ex vivo animal tissue. Our next step is to expand our training dataset with pCLE data captured from human tissue to enhance the model’s robustness to different tissue morphologies, thereby improving its generalizability. Furthermore, in our future work, the proposed probe–tissue distance regression model will be integrated with our developed orientation regression model [7]. Together, they will be deployed in our high-accuracy 6-DoF robotic system to achieve fully automated robotic tissue scanning.

## Supplementary Information

Below is the link to the electronic supplementary material.

Supplementary file 1 (pdf 490 KB)

## Footnotes

1 The dataset is available at https://zenodo.org/record/7147878#.Y2jgNux_rn5.
