# Supplementary material for: Stable distance regression via spatial–frequency state space model for robot-assisted endomicroscopy
Source: Int J Comput Assist Radiol Surg. 2025 Apr 12;20(6):1167–74. doi: 10.1007/s11548-025-03353-w (PMC12167353; doi:10.1007/s11548-025-03353-w)
Supplement: Supplementary file 1 — (pdf 490 KB) [file 11548_2025_3353_MOESM1_ESM.pdf]

Supplementary Materials

1. Contributions

In this study, our contributions are these following aspects:

- We present a novel distance regression methodology that integrates spatial and temporal information to enhance scanning stability, utilizing our newly developed Bi-directional Structured State Space model (BiS4D). This model advances traditional State Space Models by processing bi-directional image sequences and analyzing data in both frequency and spatial domains.
- we introduce a guided trajectory planning strategy that generates pseudo distance labels, facilitating the training of sequential models for smooth and stable robotic scanning trajectories.
- To improve inference speed, we also implement a Guided Fine-tune approach that efficiently reduces the BiS4D model size while maintaining performance.
- Carried out an extensive performance evaluation study where our developed regression model has been compared to state-of-the-art (SOTA) regression models. The effect of different loss functions i.e our loss function based on the Mean Absolute Error (MAE) and the Mean Absolute Percentage Error (MAPE), and the Likelihood Loss Function, has been evaluated.

2. α value ablation study

Fig.1 shows the trajectories we generated by using different values of hyper-parameter α of the Guided Trajectory Planning Strategy and how they influence the convergence speed and trajectory smoothness.

We conducted ablation experiments on the value of the hyperparameter α of the Guided Trajectory Planning Strategy and sequence length L of SF-BiS4D. Since BiS4D is based on S4D and there

are similarities between these two models, the comparison of α was tested on S4D to save time. Table 1 demonstrated that, while the model with α = 0.1 represents the optimal solution in all metrics.

Fig.1 The generated convergence trajectories corresponding to different α.

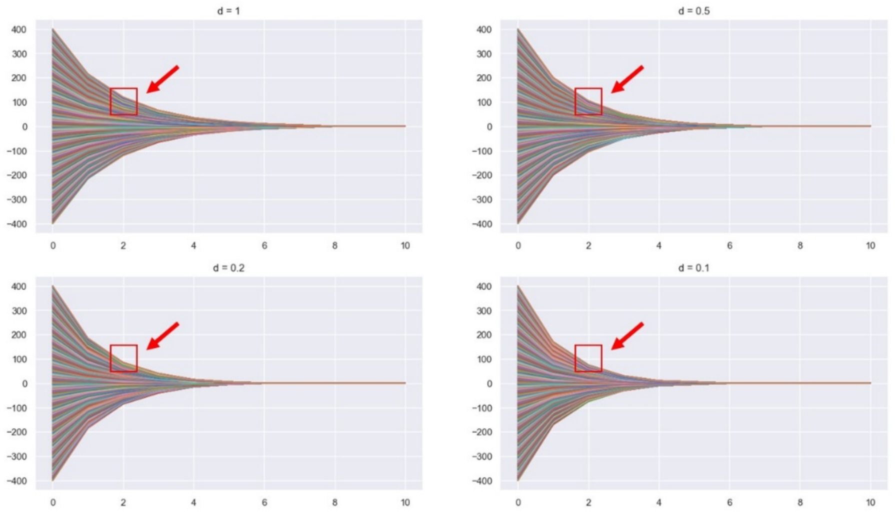

Table 1. The best results are highlighted in **bold**, and the second-best results are underlined.

α	MAE_{1st}	ACC_{dir}	MAE_{10}^{C}	MAE_{20}^{C}	BM_{10}	BM_{20}	W_{10}^{B}	W_{20}^{B}
1	**66.60**	95.36%	33.10±0.58	38.13±1.08	0.859	0.838	68.90	78.00
0.5	68.50	**95.37%**	34.50±**0.25**	39.43±1.43	0.846	0.853	**66.65**	72.81
0.2	**66.60**	95.01%	35.02±0.47	38.85±1.11	0.869	0.875	76.75	59.99
0.1	66.80	**95.37%**	**33.01±**0.47	**37.16±0.94**	**0.876**	**0.878**	70.17	**57.05**

3. Paired t-test: Statistical Significance

Table 2. Paired t-test of SOTA methods vs SF-BiS4D.

	Paired t-test of SOTA methods vs SF-BiS4D					
Metrics	SFFC-Net		DR-GAN		GA-SA-RBF	
	t	p	t	p	t	p
MAE_{C}^{20}	555.93	✓	178.67	✓	278.04	✓
BM_{20}	21.06	✓	38.79	✓	0	×
W_{20}^{B}	2603.39	✓	632.90	✓	1408.91	✓

Table 3. Paired t-test of SOTA methods vs GF-SF-BiS4D.

	Paired t-test of SOTA methods vs GF-SF-BiS4D					
Metrics	SFFC-Net		DR-GAN		GA-SA-RBF	
	t	p	t	p	t	p
MAE_{C}^{20}	718.20	✓	325.75	✓	752.50	✓
BM_{20}	21.87	✓	40.64	✓	1.85	×
W_{20}^{B}	2850.16	✓	837.52	✓	2169.13	✓

4. Evaluation Metrics

- **MAE:** The Mean Absolute Error measures the average difference between the regressed distance and the ground truth distance (lower is better). This metric indicates the

learning capacity of the model and the effectiveness of the training framework.

- $\boldsymbol{Acc_{dir}}$**:** The Moving Direction Accuracy measures the correctness of the predicted direction for the pCLE probe to approach the tissue surface, with higher values being better (higher is better). The sign of the regressed distance indicates this direction. This metric demonstrates the model's ability to guide the robot towards the tissue surface accurately.
- $\boldsymbol{MAE^C}$**:** Mean Absolute Error after convergence (MAE^C) calculates the average absolute difference between pCLE probe positions and the tissue surface upon convergence, with lower values being better. Based on the K-step incremental analysis, MAE_C is computed for both the 5th to 10th steps (MAE_{10}^C) and the 15th to 20th steps (MAE_{20}^C) after convergence. This metric indicates how closely the model guides the robot to approach the tissue surface.
- **BM:** The quality of acquired images (evaluated by blur metrics) when convergence. According to the blur metrics in [5] and the PRD dataset generation, the blur metrics are core criterion for optimal selection. Consequently, the average-normalized blur metric is used to assess the quality of the acquired pCLE images from the 5th to 10th steps (BM_{10}) and from the 15th to 20th steps (BM_{20}) .This metric directly illustrates the model's ability to facilitate the acquisition of high-quality pCLE images.
- $\boldsymbol{W^B}$**:** The Upper-Lower Bound Width (W^B) measures the difference between the upper and lower bounds of probe positions upon convergence, with lower values being better. In the testing PRD dataset, pCLE images are collected at positions ranging from -400 um to 400 um with respect to the tissue surface, at a step size of 5 um. For each collection position, there are 9-12 samples. The upper-lower bounds at each step can be calculated as follows:

$$
\begin{aligned}
\mu_p &= \frac{1}{N_p} \sum_{s \in \mathcal{S}_p} \mathcal{P}(s), \\
\mathrm{SE}_p &= \frac{1}{N_p} \sum_{s \in \mathcal{S}_p} \left| \mu_p - \mathcal{P}(s) \right|, \\
B_{up} &= \max \left\{ \mu_p + \mathrm{SE}_p \right\}_{p \in [-400,400]}, \\
B_{low} &= \min \left\{ \mu_p - \mathrm{SE}_p \right\}_{p \in [-400,400]},
\end{aligned}
$$

where, $\mathcal{P}(s)$ denotes the probe position of sample s generated by the K-step incremental analysis. The terms μ_p and SE_p represent the mean and standard error of the probe positions for the collection position p. The W^B is the difference between the maximum B_{up} and the minimum B_{low} within the specified steps. The metrics W_{10}^B and W_{20}^B are estimated at the defined steps ranging from the 5th to 10th steps and from the 15th to 20th steps, respectively. This metric reflects the model's ability to stabilize the probe upon convergence.

- $\boldsymbol{Params}$: The number of overall parameters in the model, including the frozen parameters.
